# Piecewise modelling and parameter estimation of repairable system failure rate

**DOI:** 10.1186/s40064-016-3122-4

**Published:** 2016-09-02

**Authors:** Chong Peng, Guangpeng Liu, Lun Wang

**Affiliations:** School of Mechanical Engineering and Automation, Beihang University, Beijing, 100191 China

**Keywords:** Piecewise intensity function, Reliability modelling, Parameter estimation, Artificial bee colony algorithm, Bathtub curve

## Abstract

Lifetime failure data usually presents a bathtub-shaped failure rate, and random failure phase dominates its life cycle. Relatively large errors always existed when single distribution model is used for analysis and modelling. Therefore, a bathtub-shaped reliability model based on piecewise intensity function was proposed for repairable system. Parameter estimation was studied by using maximum likelihood method in conjunction with two-stage Weibull process interval estimation and Artificial Bee Colony Algorithm. The proposed model and estimation approach fit the failure rate under bathtub curve very well, adequately reflect the duration of random failure phase, and guarantee the feasibility of quantitative analysis. Moreover, the estimated critical point of bathtub curve could be calculated. At last, the proposed model was applied in the reliability analysis of a repairable Computer Numerical Control system, and the validity of the proposed model and its parameter estimation method were verified.

## Background

Reliability modelling is a process in which the distribution curve of system failure rate is fitted based on lifetime failure data generated in system life cycle test or system operation (Myers 2010). The failure rate distribution curve can reveal system’s failure mechanism or the system-specific use phase, which may be conducive to the predication and control of failure, and carrying out an available predictive maintenance, reducing unexpected failures (Lin and Tseng 2005). Furthermore, the quantitative fitting of distribution curve is also the premise that reliability analysis, design, and test are effectively carried out. Therefore, it is important to analyze the variation trend of failure rate in a quantitative way for the purpose of reliability modelling.

At present, common distribution models for fitting failure rate include exponential distribution, normal distribution, lognormal distribution and Weibull distribution, etc. Among them, exponential distribution deals with the situation where failure rate remains unchanged, and its function is relatively simple. Weibull distribution is the most widely applied since it is able to fit the progressive increase or decrease of failure rate effectively by changing parameters. Some scholars improved exponential distribution, proposed the exponential geometric distribution (Adamidis and Loukas 1998), exponentiated exponential distribution (Gupta and Kundu 1999), the exponential-Poisson distribution (Kus 2007), the exponentiated exponential-poisson distribution (Barreto-Souza and Cribari-Neto 2009), those improved models also achieve description of the progressive increasing or decreasing of failure rate. All of which have been used monotonic function to model the failure patterns for the repairable system.

However, the lifetime failure data are often non-monotonic, and have a bathtub-shaped failure rate, which can be divided into three phases—early failure, random failure, and wear-out failure. For this reason, many scholars studied the phenomenon. Chen (2000) put forward a new two-parameter Weibull distribution model, in which the failure rate function followed the trend of bathtub curve when the shape parameter satisfied a certain condition. Later, Xie et al. (2002) and Zhang (2004) expanded the two-parameter model above by introducing the third parameter, and proposed the three-parameter Weibull distribution models independently. The results indicated that those models can reach high accuracy in fitting the trend of bathtub curve more easily than traditional models. Lai et al. (2003), based on Weibull distribution and Extreme Value Type I distribution, proposed an improved Weibull distribution model, which also described the trend of bathtub curve very well. They also completed the model’s parameter estimation by means of Weibull probability plot. El-Gohary et al. (2013) gave a new distribution known as the generalized Gompertz distribution by dealing with a new generalization of exponential. Generalized Gompertz distribution, and generalized exponential distributions, which had bathtub curve failure rate depending upon the shape parameter. And the maximum likelihood estimators of the parameters were derived using a simulations study.

Wang et al. (2015) proposed a new four parameter interval life model to describe the bathtub curve. Cordeiro et al. (2014) extended the modified Weibull distribution, and established a five-parameter Weibull distribution. Aiming at the failure censored data of industrial devices, Using Newton–Raphson type algorithm for parameter estimation, finding that the extended model fitted the bathtub curve very well.

The above models fitted the trend of bathtub-shaped failure rate well, but random failure phase was not described in detail, as well as only one critical point of early failure phase and random failure phase was included. In fact, random failure phase is the main part of life cycle for most repairable system. Single distribution model may be inadequate for analysis and modelling as well as relatively errors may occur. Parameter estimation of multi parameter model, because of the unknown parameters, is difficult to solve the problem directly by using the maximum likelihood estimation.

Therefore, a bathtub-shaped reliability model based on piecewise intensity function was proposed for repairable system. According to the characteristics of failure rate at three phases of bathtub curve, using non-homogeneous Poisson process (NHPP) and homogeneous Poisson process (HPP), the failure rate of different phases was fitted respectively. In view of the complexity of parameter estimation, relevant parameter estimation was studied by virtue of maximum likelihood method and artificial bee colony algorithm. At last, the approach was applied and verified by the reliability analysis of a repairable Computer Numerical Control (CNC) system. The remainder of the paper is organized as follows. "Set up piecewise model" section builds up a piecewise model under bathtub curve. "Parameter estimation" section studies the parameter estimation of the model. "Application" section gives an example application for verifying the proposed method. Finally, the paper is concluded.

## Set up piecewise model

Currently, there are two main methods for studying reliability of repairable system: Counting Process and Markov Process (Rausand and Høyland 2004). Markov Process is mainly targeted at the multi-state problem of repairable system. Counting Process focuses on two states which are normal operation and fault of the system, and record normal operating time of system, fault occurrence time as well as frequency, which is consistent with the trend of fault rate in this research. Consequently, Counting Process was selected for piecewise reliability modelling of trend of bathtub curve. Process is as follows:

1. Early failure phase
  At early failure phase, intensity function presents a decline trend. Due to the defects in the process of selection, manufacture and assembly of components, failure rate of system is high in the initial phase, but decreases rapidly as the run time increases and maintenance is provided. Therefore, we assume that the repair process at early failure phase is minimal repair, NHPP is used for modelling, intensity function complies with the most common Weibull process, and *b*_1_ < 1, namely:
  1 $$\omega_{1} (t) = a_{1} b_{1} t^{{b_{1} - 1}}$$
2. Random failure phase
  At random failure phase, early defects of a system have been corrected, and failure intensity is tending towards stability. Thus, we assume that the repair process at random failure phase is perfect repair, HPP and exponential distribution are adopted for modelling, and intensity function remains unchanged (as a constant):
  2 $$\omega_{2} (t) = \omega_{1} (t_{0} ) = a_{1} b_{1} t_{0}^{{b_{1} - 1}}$$
  where, *t*_0_ is the critical point between early failure phase and random failure phase.
3. Wear-out failure phase
  As run time lapses, the system slowly enters wear-out failure phase due to loss of components and mechanical fatigue, and its intensity function is increasing. Just like early failure phase, we assume that the repair process at wear-out failure phase is minimal repair, NHPP is used for modelling, and intensity function complies with Weibull process. Thus, the system’s intensity function is:
  3 $$\omega_{3} (t) = a_{1} b_{1} t_{0}^{{b_{1} - 1}} + a_{2} b_{2} (t - t_{1} )^{{b_{2} - 1}}$$
  where, *t*_1_ is the critical point between random failure phase and wear-out failure phase.

To sum up, the system’s intensity function throughout the life cycle is:

4 $$\omega (t) = \left\{ {\begin{array}{*{20}l} {a_{1} b_{1} t^{{b_{1} - 1}} } \hfill & \quad {t \le t_{0} } \hfill \\ {a_{1} b_{1} t_{0}^{{b_{1} - 1}} } \hfill & \quad {t_{0} < t \le t_{1} } \hfill \\ {a_{1} b_{1} t_{0}^{{b_{1} - 1}} + a_{2} b_{2} (t - t_{1} )^{{b_{2} - 1}} } \hfill & \quad {t > t_{1} } \hfill \\ \end{array} } \right.$$

Then, the system’s cumulative intensity function is:

5 $$W_{c} (t) = \int_{0}^{t} {\omega (u)du} = \left\{ {\begin{array}{*{20}l} {a_{1} t^{{b_{1} }} } \hfill & \quad {t \le t_{0} } \hfill \\ {a_{1} t_{0}^{{b_{1} }} + a_{1} b_{1} t_{0}^{{b_{1} - 1}} (t - t_{0} )} \hfill & \quad {t_{0} < t \le t_{1} } \hfill \\ {a_{1} t_{0}^{{b_{1} }} + a_{1} b_{1} t_{0}^{{b_{1} - 1}} (t - t_{0} ) + a_{2} (t - t_{1} )^{{b_{2} }} } \hfill & \quad {t > t_{1} } \hfill \\ \end{array} } \right.$$

## Parameter estimation

There are a lot of methods for parameter estimation of reliability model, such as maximum likelihood estimation, moment estimation, least square method, and Bayesian method. Among these methods, maximum likelihood estimation is the most widely used owing to its good theoretical basis and high estimation accuracy. Therefore, maximum likelihood estimation was applied for parameter estimation of the proposed reliability model.

### Maximum likelihood estimation

First, the likelihood function shall be determined. Based on the reliability model above, the cumulative distribution function of the *i*th Mean Time Between Failure (MTBF) is:

6 $$\begin{aligned} F(S_{i} \left| {S_{i - 1} } \right.) & = \frac{{F(S_{i} ) - F(S_{i - 1} )}}{{1 - F(S_{i - 1} )}} \\ & = \frac{{\exp [ - W(S_{i - 1} )] - \exp [ - W(S_{i} )]}}{{\exp [ - W(S_{i - 1} )]}} \\ & = 1 - \exp [ - W(S_{i} ) + W(S_{i - 1} )] \\ \end{aligned}$$

where, *S*_*i*_ is the moment when the *i*th failure occurs. Then, the conditional probability density function of *S*_*i*_ is:

7 $$f\left( {S_{i} \left| {S_{i - 1} } \right.} \right) = \omega (S_{i} )\exp \left[ { - W(S_{i} ) + W(S_{i - 1} )} \right]$$

Equations (4) and (5) are plugged into (7), and we obtain: When *S*_*i*_ ≤ *t*_0_,

8 $$f(S_{i} \left| {S_{i - 1} } \right.) = a_{1} b_{1} S_{i}^{{b_{1} - 1}} \exp \left( { - a_{1} S_{i}^{{b_{1} }} + a_{1} S_{i - 1}^{{b_{1} }} } \right)$$

When *S*_*i*−1_ < *t*_0_ ≤ *S*_*i*_ < *t*_1_,

9 $$f(S_{i} \left| {S_{i - 1} } \right.) = a_{1} b_{1} t_{0}^{{b_{1} - 1}} \exp \left[ { - a_{1} (1 - b_{1} )t_{0}^{{b_{1} }} - a_{1} b_{1} t_{0}^{{b_{1} - 1}} S_{i} + a_{1} S_{i - 1}^{{b_{1} }} } \right]$$

When *t*_0_ < *S*_*i*−1_ < *S*_*i*_ ≤ *t*_1_,

10 $$f(S_{i} \left| {S_{i - 1} } \right.) = a_{1} b_{1} t_{0}^{{b_{1} - 1}} \exp \left[ { - a_{1} b_{1} t_{0}^{{b_{1} - 1}} (S_{i} - S_{i - 1} )} \right]$$

When *S*_*i* −1_ < *t*_1_ < *S*_*i*_,

11 $$f(S_{i} \left| {S_{i - 1} } \right.) = \left[ {a_{1} b_{1} t_{0}^{{b_{1} - 1}} + a_{2} b_{2} (S_{i} - t_{1} )^{{b_{2} - 1}} } \right]\exp \left[ { - a_{1} b_{1} t_{0}^{{b_{1} - 1}} (S_{i} - S_{i - 1} ) - a_{2} (S_{i} - t_{1} )^{{b_{2} }} } \right]$$

When *t*_1_ < *S*_*i*-1_ < *S*_*i*_,

12 $$\begin{aligned} f(S_{i} \left| {S_{i - 1} } \right.) & = \left[ {a_{1} b_{1} t_{0}^{{b_{1} - 1}} + a_{2} b_{2} (S_{i} - t_{1} )^{{b_{2} - 1}} } \right] \\ & \quad \times \exp \left[ { - a_{1} b_{1} t_{0}^{{b_{1} - 1}} (S_{i} - S_{i - 1} ) \, - a_{2} (S_{i} - t_{1} )^{{b_{2} }} + a_{2} (S_{i - 1} - t_{1} )^{{b_{2} }} } \right] \\ \end{aligned}$$

When the system’s failure time data is Type-II censored data, the likelihood function is:

13 $$\begin{aligned} L(S_{1} ,S_{2} , \ldots ,S_{n} \left| \theta \right.) & = \prod\limits_{i = 1}^{n} {f(S_{i} \left| {S_{i - 1} } \right.)} \\ & = \prod\limits_{i = 1}^{k} {a_{1} b_{1} S_{i}^{{b_{1} - 1}} \exp \left( { - a_{1} S_{i}^{{b_{1} }} + a_{1} S_{i - 1}^{{b_{1} }} } \right)} \times a_{1} b_{1} t_{0}^{{b_{1} - 1}} \\ & \quad \times \exp \left[ { - a_{1} (1 - b_{1} )t_{0}^{{b_{1} }} - a_{1} b_{1} t_{0}^{{b_{1} - 1}} S_{k + 1} + a_{1} S_{k}^{{b_{1} }} } \right] \\ & \quad \times \prod\limits_{i = k + 2}^{l} {a_{1} b_{1} t_{0}^{{b_{1} - 1}} \exp [ - a_{1} b_{1} t_{0}^{{b_{1} - 1}} (S_{i} - S_{i - 1} )]} \\ & \quad \times \left[ {a_{1} b_{1} t_{0}^{{b_{1} - 1}} + a_{2} b_{2} (S_{l + 1} - t_{1} )^{{b_{2} - 1}} } \right] \\ & \quad \times \exp \left[ { - a_{1} b_{1} t_{0}^{{b_{1} - 1}} (S_{l + 1} - S_{l} ) - a_{2} (S_{l + 1} - t_{1} )^{{b_{2} }} } \right] \\ & \quad \times \prod\limits_{i = l + 2}^{n} {\left\{ {\left[ {a_{1} b_{1} t_{0}^{{b_{1} - 1}} + a_{2} b_{2} (S_{i} - t_{1} )^{{b_{2} - 1}} } \right]} \right.} \\ & \quad \left. { \times \exp \left[ { - a_{1} b_{1} t_{0}^{{b_{1} - 1}} (S_{i} - S_{i - 1} ) - a_{2} (S_{i} - t_{1} )^{{b_{2} }} + a_{2} (S_{i - 1} - t_{1} )^{{b_{2} }} } \right]} \right\} \\ \end{aligned}$$

where *n* is the total number of failures, *θ* = *(a*1*, b*1*, t*0*, a*2*, b*2*, t*1*, ρ), S*_*k*_ < *t*_0_ < *S*_*k*+1_ < ⋯ < *S*_*l*_ < *t*_1_ < *S*_*l*+1_.

When the system’s failure time data is Type-I censored data, the likelihood function is:

14 $$L(S_{1} ,S_{2} , \ldots ,S_{n} \left| \theta \right.) = \prod\limits_{i = 1}^{n} {f(S_{i} \left| {S_{i - 1} } \right.)} R(t_{s} \left| {S_{n} } \right.)$$

Where *t*_*s*_ is censoring time,

15 $$\begin{aligned} R(t_{s} \left| {S_{n} } \right.) & = \exp [ - W_{c} (t_{s} ) + W_{c} (S_{n} )] \\ & = \exp [ - a_{1} b_{1} t_{0}^{{b_{1} - 1}} (t_{s} - S_{n} ) - a_{2} (t_{s} - t_{1} )^{{b_{2} }} + a_{2} (S_{n} - t_{1} )^{{b_{2} }} ] \\ \end{aligned}$$

To sum up, the likelihood function is:

16 $$L(S_{1} ,S_{2} , \ldots ,S_{n} \left| \theta \right.) = \prod\limits_{i = 1}^{n} {f(S_{i} \left| {S_{i - 1} } \right.)} \delta R(t_{s} \left| {S_{n} } \right.)$$

Where *δ* is defined as:

17 $$\delta = \left\{ {\begin{array}{*{20}l} 0 \hfill & \quad {{\text{Type-II}}\,{\text{censored}}\,{\text{data}}} \hfill \\ 1 \hfill & \quad {{\text{Type I}}\,{\text{censored}}\,{\text{data}}} \hfill \\ \end{array} } \right.$$

Given that the specific equation of likelihood function is related to the interval of failure time data where *t*_0_ and *t*_1_ are located, and it is difficult to solve the complicated equation of likelihood function by calculating logarithmic derivative. Hence, artificial bee colony algorithm was used for parameter optimization to maximize likelihood in accordance with the piecewise processing of *t*_0_ and *t*_1_. The optimization model is as follows:

18 $$\begin{aligned} & \mathop{\hbox{max} }\limits_{\theta } \quad L(S_{1} ,S_{2} , \ldots ,S_{n} \left| \theta \right.) \\ & {\text{subject}}\,{\text{to}}\;\left\{ {\begin{array}{*{20}l} {a_{1} > 0,0 < b_{1} \le 1} \hfill \\ {a_{2} > 0,b_{2} \ge 1} \hfill \\ {0 < t_{0} < t_{1} < S_{n} } \hfill \\ \end{array} } \right. \\ \end{aligned}$$

In addition, the range of variable parameter shall be optimized to improve efficiency and accuracy of the model’s parameter optimization. First, the value range of *t*_0_ and *t*_1_ *, i.e. k* and *l*, shall be determined based on the system’s failure time data and trend estimation. Second, the first *k* time data at early failure phase and the last *n*–*l* time data at wear-out failure phase are adopted for interval estimation of Weibull process, and the extreme value of estimation interval is regarded as the optimization range of parameters *a*_1_ *, b*_1_ *, a*_2_, and *b*_2_.

**Fig. 1:**
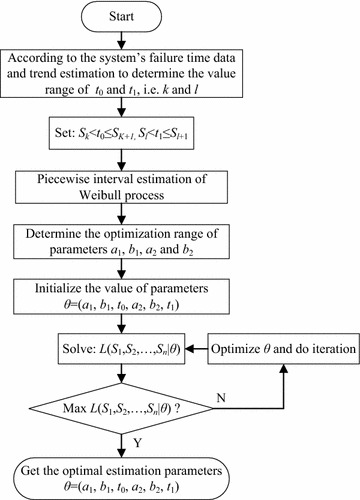
Flowchart of parameter estimation of reliability model

The flowchart of parameter estimation of reliability model is shown in Fig. 1.

### Weibull process interval estimation

Interval estimation includes two parts—point estimation and margin of error that describes estimation accuracy. Therefore, to complete the interval estimation of Weibull process, the point estimation of parameters shall be first performed, followed by the calculation of its margin of error.

1. Point estimation
  Based on Literature (Ebeling 2004), the maximum likelihood point estimation of Weibull process parameters *a* and *b* is as follows:
  When the failure time data is Type-II censored data:
  19 $$\left\{ {\begin{array}{*{20}l} {\hat{b} = \frac{n}{{\sum\nolimits_{i = 1}^{n} {\ln \frac{{S_{n} }}{{S_{i} }}} }}} \hfill \\ {\hat{a} = \frac{n}{{S_{n}^{{\hat{b}}} }}} \hfill \\ \end{array} } \right.$$
  When the failure time data is Type-I censored data:
  20 $$\left\{ {\begin{array}{*{20}l} {\hat{b} = \frac{n}{{\sum\nolimits_{i = 1}^{n} {\ln \frac{{t_{s} }}{{S_{i} }}} }}} \hfill \\ {\hat{a} = \frac{n}{{t_{s}^{{\hat{b}}} }}} \hfill \\ \end{array} } \right.$$
2. Margin of error
  Generally, in the case of large sample (≥30), the maximum likelihood point estimation presents consistency and asymptotically normal distribution; In the case of small sample, the logarithm of point estimation of parameter is much closer to normal distribution. Specific equations are as follows:
  In the case of large sample:
  21 $$\frac{{a - \hat{a}}}{{\sqrt {D(\hat{a})} }} \sim N(0,1);\quad \frac{{b - \hat{b}}}{{\sqrt {D(\hat{b})} }} \sim N(0,1)$$
  In the case of small sample:
  22 $$\frac{{\ln a - \ln \hat{a}}}{{\sqrt {D(\hat{a})} }} \sim N(0,1);\quad \frac{{\ln b - \ln \hat{b}}}{{\sqrt {D(\hat{b})} }} \sim N(0,1)$$
  where $D(\hat{a})$ and $D(\hat{b})$ are the variance of parameters *a* and *b*, respectively. Their value can be obtained using Fisher Information Matrix below (Kijima 1989; Ye 2003):
  23 $$\left( {\begin{array}{*{20}l} {D(\hat{a})} \hfill & \quad {\text{cov} (\hat{a},\hat{b})} \hfill \\ {\text{cov} (\hat{b},\hat{a})} \hfill & \quad {D(\hat{b})} \hfill \\ \end{array} } \right) = \left( {\begin{array}{*{20}l} { - \frac{{\partial^{2} \ln L}}{{\partial a^{2} }}} \hfill & \quad { - \frac{{\partial^{2} \ln L}}{\partial a\partial b}} \hfill \\ { - \frac{{\partial^{2} \ln L}}{\partial b\partial a}} \hfill & \quad { - \frac{{\partial^{2} \ln L}}{{\partial b^{2} }}} \hfill \\ \end{array} } \right)^{ - 1}$$

According to normal distribution, the confidence interval under 1−*α*:

In the case of large sample:

24 $$ P\left( {\frac{{\left| {a - \hat{a}} \right|}}{{\sqrt {D(\hat{a})} }} \le Z_{{\alpha /2}} } \right) = 1 - \alpha ;\quad P\left( {\frac{{\left| {b - \hat{b}} \right|}}{{\sqrt {D(\hat{b})} }} \le Z_{{\alpha /2}} } \right) = 1 - \alpha $$

In the case of small sample:

25 $$ P\left( {\frac{{\left| {\ln a - \ln \hat{a}} \right|}}{{\sqrt
{D(\hat{a})} }} \le Z_{{\alpha /2}} } \right) = 1 - \alpha ;\quad
P\left( {\frac{{\left| {\ln b - \ln \hat{b}} \right|}}{{\sqrt
{D(\hat{b})} }} \le Z_{{\alpha /2}} } \right) = 1 - \alpha $$

Solving Eq. (25) can obtain the confidence interval for *a* and *b*:

In the case of large sample:

26 $$ \left[ {\hat{a} - Z_{{\alpha /2}} \sqrt {D(\hat{a})} , \, \hat{a} +
Z_{{\alpha /2}} \sqrt {D(\hat{a})} } \right];\quad \left[ {\hat{b} -
Z_{{\alpha /2}} \sqrt {D(\hat{b})} , \, \hat{b} + Z_{{\alpha /2}}
\sqrt {D(\hat{b})} } \right] $$

In the case of small sample:

27 $$ \left[ {\hat{a}/\exp \left( {Z_{{\alpha /2}} \sqrt {D(\hat{a})} } \right),\,\hat{a} \cdot \exp \left( {Z_{{\alpha /2}} \sqrt {D(\hat{a})} } \right)} \right];\quad \left[ {\hat{b}/\exp \left( {Z_{{\alpha /2}} \sqrt {D(\hat{b})} } \right),\,\hat{b} \cdot \exp \left( {Z_{{\alpha /2}} \sqrt {D(\hat{b})} } \right)} \right] $$

### Parameter optimization based on artificial bee colony algorithm

With respect to the proposed model for parameter optimization and the range optimized by Weibull process interval estimation, artificial bee colony algorithm was used for solving optimization problem. Artificial bee colony algorithm is characterized by strong global optimization, rapid rate of convergence, and suitability in solving different problems, compared with other swarm intelligence optimization algorithms such as evolutionary algorithm, artificial immune algorithm, particle swarm optimization, and ant colony algorithm (Chen 2015).

The correspondence between bee’s search for nectar source and optimization in artificial bee colony algorithm is shown in Table 1.

**Table 1:** Correspondence between bee’s behavior and optimization

Bee searches for nectar source	Optimization
Location of nectar source	Value of all *θ* = (*a*_1_, *b*_1_, *t*_0_, *a*_2_, *b*_2_, *t*_1_)
Quality of nectar source	Fitness value corresponding to all θ, i.e. L(S_1_,S_2_,…,S_n_|θ)
Speed of gathering honey	Solving speed
Maximum fitness	*max L*(*S*_1_,*S*_2_,…,*S*_*n*_|*θ*)

The algorithm process is as follows:

1. Initialization of parameter
  First, the two basic parameters in artificial bee colony algorithm shall be initialized. One parameter is the quantity of nectar source (*S*_*n*_), which represents the number of solution. Besides, *S*_*n*_ is also the number of leaders and followers; the other parameter is the *limit* value of gathering nectar source (*limit*). When the gathering times of a nectar source exceed the *limit*, the nectar source will be abandoned.
2. Initialization of solution space
  Random initialization produces the primary *S*_*n*_ solutions. Let *θ*_*i*_ = *(a*_1*i*_ *, b*_1*i*_ *, t*_0*i*_ *, a*_2*i*_ *, b*_2*i*_ *, t*_1*i*_ *)* = *(θ*_*i*1_ *, θ*_*i*2_ *, θ*_*i*3_ *, θ*_*i*4_ *, θ*_*i*5_ *, θ*_*i*6_ *)*, which represents the location of the *i*th nectar source. The initialization formula for each value is as follows:
  28 $$\theta_{ij} = LB_{j} + (UB_{j} - LB_{j} ) \times r\quad j = 1,2, \ldots ,4\quad i = 1,2, \ldots ,Sn$$
  where *r* is the random number between [0, 1], and *LB*_*j*_ and *UB*_*j*_ are the value range of *θ*_*j*_, i.e. minimum and maximum.
  Later, the leaders start to gather these nectar sources at random, and relevant fitness value is calculated.
3. Stage of leaders
  When the leaders decide to gather a nectar source at random, they will search new nectar sources at random around the nectar source. Their search is in line with the equation below:
  29 $$\theta_{new(j)} = \theta_{ij} + (\theta_{kj} - \theta_{ij} ) \times r\quad k \in (1,2, \ldots ,Sn) \cap k \ne i\quad j = 1,2, \ldots ,4$$
  The fitness value corresponding to new nectar sources *θ*_*new*_ is then calculated and compared with previous nectar sources to select the solution with higher fitness.
4. Stage of followers
  In terms of followers, their follow probability is calculated based on normalization and fitness value of each nectar source. The equation is as follows:
  30 $$P_{i} = \frac{{fit_{i} }}{{\sum\nolimits_{i = 1}^{Sn} {fit_{i} } }}$$
  where, *fit*_*i*_ represents the fitness value of nectar source at *θ*_*i*_. Larger *fit*_*i*_ indicates greater probability that the followers select the nectar source. When the followers select a nectar source, they also search new nectar sources at random around the nectar source based on Eq. (29) just as the leaders do, and then choose a better nectar source by comparing with the nectar source they follow.
5. Stage of scouters
  At this stage, if a nectar source *θ*_*i*_ is still not improved after being gathered *limit* times, it will be abandoned. The leaders here will become scouters, and search a new nectar source at random based on Eq. (28).
6. Iterations
  The nectar source with the maximum fitness is recorded. Whether iterations reach the maximum set point is judged. If the maximum set point is reached, the algorithm ends and the optimal nectar source, namely optimal solution, is output. Otherwise, return to (3) to continue the cycle.

## Application

The proposed model was applied in reliability analysis of a repairable CNC system containing servo drive unit.

**Fig. 2:**
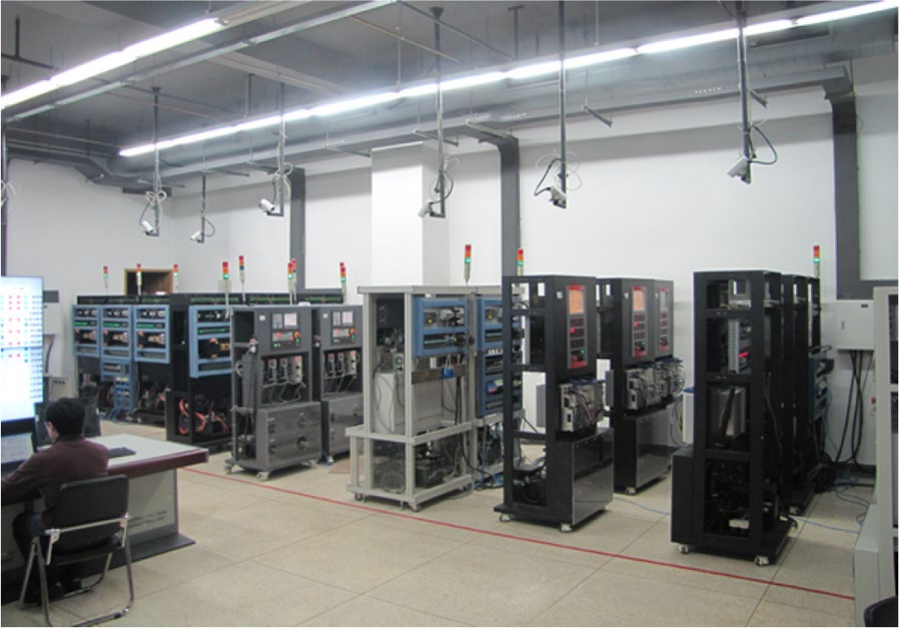
The environment of CNC system reliability test laboratory

In order to test the reliability of CNC system, a long-term multi-sample test (Type-I censored) under the same environment was carried out, and the environment of test laboratory as shown in Fig. 2. This test has been taken to record the failures of CNC system in the past two years. The failure time data of 18 sets of CNC system with 50 failures were recorded, and processed by the Total Test Time method (Barlow and Campo 1975), as shown in Table 2.

**Table 2:** Data of total failure time

Number of failure	Total failure time (h)	Number of failure	Total failure time (h)	Number of failure	Total failure time (h)	Number of failure	Total failure time (h)
1	216	14	53,262	27	129,726	40	191,921
2	522	15	58,536	28	132,588	41	193,253
3	990	16	66,546	29	139,770	42	195,282
4	1368	17	68,310	30	145,314	43	197,686
5	4248	18	76,734	31	149,868	44	199,193
6	6354	19	80,406	32	153,054	45	199,901
7	10,728	20	85,842	33	162,036	46	200,671
8	17,046	21	95,400	34	168,666	47	201,244
9	23,652	22	97,578	35	174,137	48	201,739
10	29,862	23	107,010	36	179,169	49	202,810
11	35,730	24	111,492	37	183,413	50	202,978
12	39,276	25	116,892	38	186,045	Type-I censored test time: ts = 203,115	
13	48,366	26	121,662	39	189,225		

**Fig. 3:**
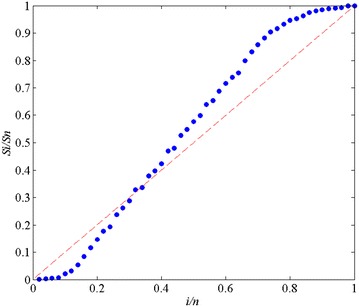
TTT diagram of system failure time data

Based on the data above, the system’s failure trend estimation and test were carried out, and TTT diagram (Bergman 1979) was drawn, as shown in Fig. 3. Where, the total number of failures *n* = 50.

As can be seen in Fig. 3, the TTT scatter diagram is concave under the diagonal of unit square at the beginning, indicating that the failure rate presents a decline at early phase. Later, the diagram fluctuates slightly along a straight line. The failure rate remains unchanged, and the system is gradually stabilized. After a period of time, the diagram looks like a convex under the diagonal of unit square, indicating that the failure rate increases.

Meanwhile, Laplace test (Louit et al. 2009) and Anderson–Darling test (Pulcini 2001) were used for trend test of failure data in Table 2. The significance level was set as *α* = 0.05. The results of statistical tests are shown in Table 3.

**Table 3:** Results of statistical tests at the significance level *α* = 0.05

Test methods	Statistics	Critical value	Calculated value	Results
Laplace test	*U*	1.96	1.35	Show a non-monotonic trend
Anderson–Darling test	*V*_1_	1.96	3.32	Follow the trend of bathtub curve
	*V*_2_	1.96	4.08	
	*V*_3_	192.47	68.65	

The results of trend test are consistent with the estimation output of TTT diagram. The system failure time data follows the trend of bathtub curve, and undergoes three phases—early failure, random failure and wear-out failure.

Then, the system reliability model was set up using the piecewise function above, and parameter estimation was performed.

First, the optimum ranges of *t*_0_ and *t*_1_ were processed in segment, and within the scope of (0, *n*) select the values of *k* and *l* in turn. But in order to improve calculation efficiency, by observing the total failure time data and the inflection point trend of TTT diagram, the range of values for *k* and *l* can be reduced and make a preliminary judgment. So, the value of *k* may be 6, 7, 8, and 9, while the value of *l* may be 33, 34, 35, and 36. And, the value range of *t*_0_ and *t*_1_ is *S*_*k*_ < *t*_0_ < *S*_*k*+1_ < *S*_*l*_ < *t*_1_ < *S*_*l*+1_.

Second, parameter optimization was conducted in accordance with different *k* and *l*. Based on the failure time sequence *S*_1_ *, S*_2_ *, S*_3_ *,…, S*_*k*_ (considered as Type-II censored data), interval estimation of minimal repair model at early failure phase was conducted. In the condition that the given confidence was 99.73 %, the confidence interval was *a*_1_ ∈ [*a*_1*min*_ *, a*_1*max*_], *b*_1_ ∈ [*b*_1*min*_ *, b*_1*max*_]. Similarly, based on the failure time sequence *S*_*l*+1_ *,…, S*_50_ *, t*_*s*_ (considered as Type-I censored data), interval estimation of minimal repair model at wear-out failure phase was conducted. In the condition that the given confidence was 99.73 %, the confidence interval was *a*_2_ ∈ [*a*_2*min*_ *, a*_2*max*_], *b*_2_ ∈ [*b*_2*min*_ *, b*_2*max*_]. The improved optimization model was:

31 $$\begin{aligned} & \mathop {\text{max} }\limits_{\theta } L(S_{1} ,S_{2} , \ldots ,S_{n} \left| \theta \right.) \\ & {\text{subject}}\,{\text{to}}\,\left\{ {\begin{array}{*{20}l} {a_{1{\text{min}} } \le a_{1} \le a_{1{\text{max}} } ,b_{1{\text{min}} } \le b_{1} \le b_{1{\text{max}} } } \hfill \\ {a_{2{\text{min}} } \le a_{2} \le a_{2{\text{max}} } ,b_{2{\text{min}} } \le b_{2} \le b_{2{\text{max}} } } \hfill \\ {S_{k} < t_{0} < S_{k + 1} ,S_{l} < t_{1} < S_{l + 1} } \hfill \\ \end{array} } \right. \\ \end{aligned}$$

Artificial bee colony algorithm was then used for parameter optimization of the optimization model above. The algorithm’s iterations, size of bee colony, and *limit* were set. The optimal parameter in the case of *k* and *l* was *θ* = *(a*_1_ *, b*_1_ *, t*_0_ *, a*_2_ *, b*_2_ *, t*_1_ *)*.

At last, optimal parameters in the case of different *k* and *l* were compared. The parameter that maximizes likelihood was selected as the optimal parameter of overall model, i.e. *k* = 7, *l* = 33, *a*_1_ = 0.0427, *b*_1_ = 0.5066, *t*_0_ = 16,567, *a*_2_ = 5.0503 × 10–11, *b*_2_ = 2.4984, *t*_1_ = 168,242.

The goodness of fit of the two-stage Weibull process above was tested using Cramer-Von Mises test (Ebeling 2004). The given significance level was *α* = 0.05. The test results are shown in Table 4.

**Table 4:** Results of test of goodness of fit at the significance level *α* = 0.05

Weibull process	Test methods	Statistics	Critical value	Calculated value	Results
Stage I	Cramer-Von Mises test	$C_{M}^{2}$	0.208	0.079	Verified
Stage II			0.217	0.151	Verified

In conclusion, 18 sets of CNC system intensity functions were obtained:

32 $$\omega (t) = \left\{ {\begin{array}{*{20}l} {0.0216 \times t^{ - 0.4934} } \hfill & \quad {t \le 16567} \hfill \\ { 1. 7 8 9 3\times 1 0^{ - 4} } \hfill & \quad { 16567< t \le 168242} \hfill \\ { [ 1.7893\times 1 0^{ - 4} + 1.2618 \times 10^{ - 10} \times (t - 168242)^{1.4984} } \hfill & \quad {t > 168242} \hfill \\ \end{array} } \right.$$

Meanwhile, the results suggest that the critical point between early failure phase and random failure phase of a single system was located at about *t*_0_/18 = 920 h, while the critical point between random failure phase and wear-out failure phase was located at about *t*_1_/18 = 9347 h.

The intensity function of a single system was:

33 $$\omega_{1} (t) = \left\{ {\begin{array}{*{20}l} {5.1882 \times 10^{ - 3} \times t^{ - 0.4934} } \hfill & \quad {t \le 920} \hfill \\ { 1.7893\times 1 0^{ - 4} } \hfill & \quad { 920< t \le 9347} \hfill \\ { [ 1.7893\times 1 0^{ - 4} + 9.5916 \times 10^{ - 9} \times (t - 9347)^{1.4984} } \hfill & \quad {t > 9347} \hfill \\ \end{array} } \right.$$

**Fig. 4:**
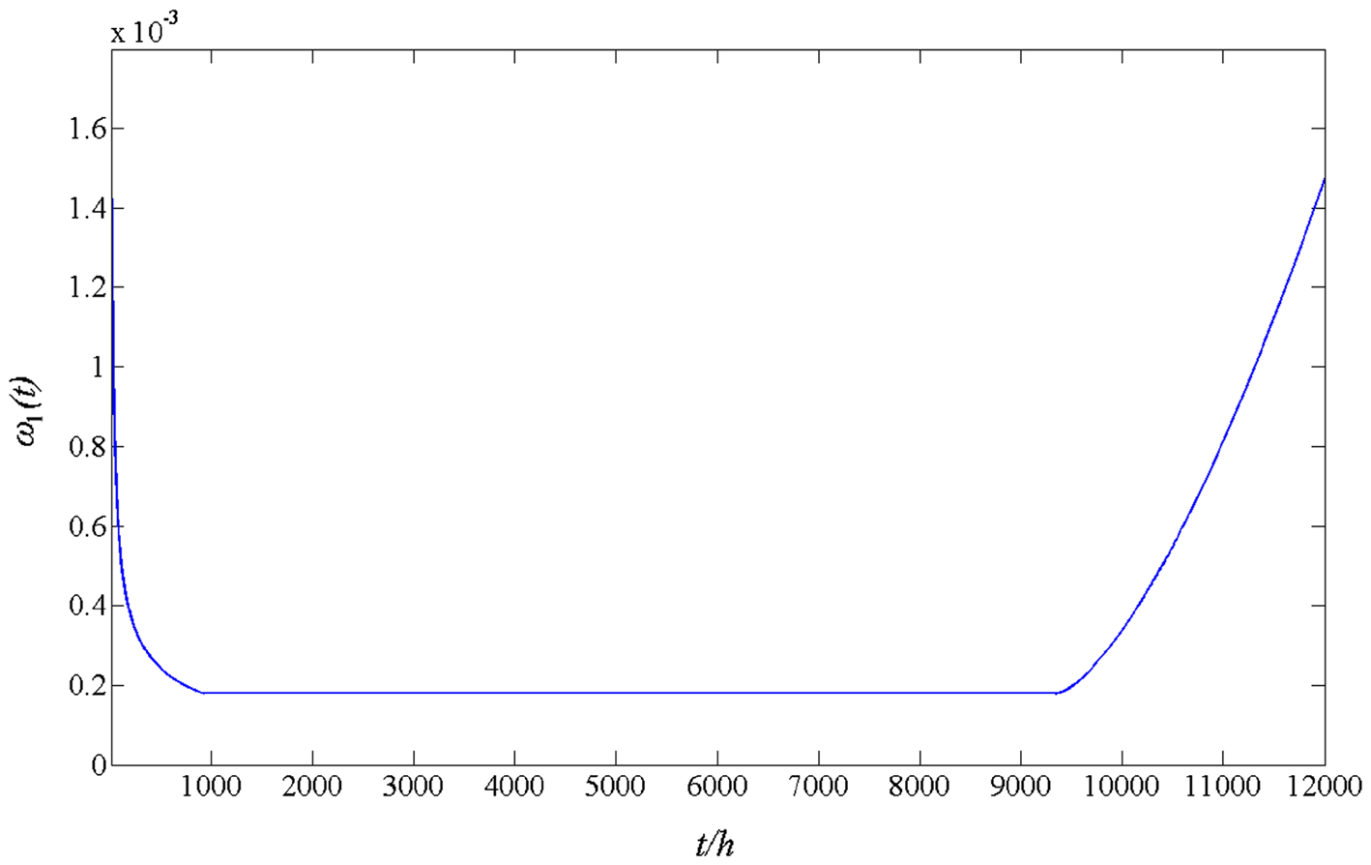
Intensity function of a single system

The curve of intensity function is shown in Fig. 4.

Based the instantaneous MTBF(*t*) = 1/ω(*t*), we found that the MTBF of the system at random failure phase was calculated as MTBF = 1/(1.7893 × 10^−4^) = 5589 h.

Therefore, the example above proves that the proposed piecewise reliability model can fit the failure rate under bathtub curve very well and obtain two critical points of bathtub curve. It provides an important basis for reliability analysis, design and test.

## Conclusions

A modelling study on the phenomenon that the failure rate of lifetime failure data presents a bathtub curve was carried out for repairable system. The following achievements were made:

1. Considering the characteristics of failure rate at three phases of bathtub curve, and combining homogeneous Poisson process and non-homogeneous Poisson process during Counting Process, a bathtub-shaped reliability model based on piecewise intensity function was proposed. Moreover, with respect to the difficulty of common parameter estimation methods in solving equations, maximum likelihood estimation and artificial bee colony algorithm were combined to estimate the model-related parameters and guarantee the feasibility of the model in application.
2. Given that extensive range and excessive time consumption of parameter optimization during parameter estimation, the interval estimation of two-stage Weibull process was studied, and the range of model optimization was improved. As a result, the efficiency of parameter optimization was substantially increased.
3. The proposed model, as well as estimation approach, fits the failure rate under bathtub curve very well and adequately reflects the duration of random failure phase. Besides, two critical points of bathtub curve are obtained.
4. At last, the practical use of the proposed model is demonstrated by a set of failure data of a repairable CNC system, and the validity of the proposed model and its parameter estimation method was verified. The study will be useful for reliability analysis of repairable systems and provide an important basis for relevant reliability research.
